# Dr. Ch. Robin, of Paris

**Published:** 1857-10

**Authors:** 


*Dr. Ch. Robin, of Paris.*—We find in a letter of the Parisian correspondent of the New York Times, a glowing but just tribute to Robin. Of the medical luminaries of Paris, he is a "bright particular star." To listen to his instructions; to see and know him and kindle one's own zeal by witnessing his enthusiasm and self-sacrificing industry, are objects of themselves, sufficient to repay for crossing the Atlantic. The labors of Robin are not not known in this country so much as their importance claims. His great work incorrectly styled anatomical and physiological chemistry, prepared in conjunction with M. Verdeil, is yet to be translated. He has been for some years past engaged on a still larger work—general anatomy, healthy and morbid—which we trust will soon be completed. It is safe to predict that the publication of this work will form an important epoch in the history of these branches of medical science. By his admirers, Robin is often styled the Bichat of the present day. The following is the passage in the letter referred to: *

"There is a young physician at Paris, whose example is well worthy a notice here. His is a name which is heard hundreds of times daily from one end of Europe to the other in the mouths of the most distinguished men of science of all countries. And yet he is a poor man, who dines at a cheap restaurant in the Latin quarter with students, and who lives upon a patrimony that would scarcely pay the servant hire of many of his colleagues in science. This is Robin, the microscopist. He is a deathly pale, thin, serious-looking young man, of about thirty-four years of age. His whole life is devoted, by means of the microscope, to the study, the demonstration and classification of morbid tissues. There is scarcely a cancer excised at Paris, nor a doubtful *post-mortem* examination made, that Robin and his microscope are not consulted, and his word is authority. His whole life is spent in the exploration of the dead body in order to benefit the living. And all this he does modestly, in poverty, and to the sacrifice of his health, for the promotion of pure science and correct opinions. He has, it is true, the gratification of being adored by his colleagues, old and yoxng, of never having his name pronounced but with veneration; but it is such men as these that are neglected by the public."

